# On the Deployment of a Connected Sensor Network for Confident Information Coverage

**DOI:** 10.3390/s150511277

**Published:** 2015-05-14

**Authors:** Huping Xu, Jiajun Zhu, Bang Wang

**Affiliations:** 1 School of Logistics Engineering, Wuhan University of Technology, Heping Road #1040, Wuhan 430070, China; E-Mail: hupingxu@126.com; 2 School of Electronic, Information and Communications, Huazhong University of Science and Technology, Luoyu Lu #1037, Wuhan 430074, China; E-Mail: zhu5034@gmail.com

**Keywords:** wireless sensor networks, confident information coverage, connectivity, sensor placement problem

## Abstract

Coverage and connectivity are two important performance metrics in wireless sensor networks. In this paper, we study the sensor placement problem to achieve both coverage and connectivity. Instead of using the simplistic disk coverage model, we use our recently proposed confident information coverage model as the sensor coverage model. The grid approach is applied to discretize the sensing field, and our objective is to place the minimum number of sensors to form a connected network and to provide confident information coverage for all of the grid points. We first formulate the sensor placement problem as a constrained optimization problem. Then, two heuristic algorithms, namely the connected cover formation (CCF) algorithm and the cover formation and relay placement with redundancy removal (CFRP-RR) algorithm, are proposed to find the approximate solutions for the sensor placement problem. The simulation results validate their effectiveness, and the CCF algorithm performs slightly better than the CFRP-RR algorithm.

## 1. Introduction

A wireless sensor network (WSN) consists of a large number of spatially-distributed, low-cost sensors to monitor and gather information about various physical phenomena within the sensing field. Wireless sensor networks are an active research area in computer science and telecommunication and have been widely used in a variety of applications nowadays, such as precision agriculture, environment sensing, military surveillance, and so on [1–3].

Coverage is an important performance metric in WSNs, which reflects how well a sensing field is monitored [4]. We may interpret the coverage concept as a nonnegative mapping between the space points of a sensing field and the sensors of a wireless sensor network. We usually use the sensor coverage model to measure the sensing capability and quality of a sensor. The widely-used disk coverage model assumes that a sensor can cover a disk centered on itself with the radius of its sensing range. The disk coverage model, however, is a simplistic sensor coverage model, which does not consider the spatial correlation of physical phenomena and information processing paradigm via the collaboration of sensors. Motivated by the precision agriculture applications [5,6] and based on the theory of field reconstruction [7], we have proposed a new sensor coverage model, called confident information coverage (CIC or Φ-coverage), in our previous study [8]. We will briefly introduce the CIC model in Section 3.

Connectivity is another important performance metric in WSNs, which is a sufficient condition for reliable information transmission. Due to the limited energy, sensors can only transmit information at a short distance. In this paper, we assume a disk communication model: each sensor has a communication range of *R_c_*, and any two sensors can communicate with each other if and only if the Euclidean distance between them is not larger than *R_c_*. A long-distance information transmission is only possible through hop-by-hop information forwarding. A WSN is called connected if and only if any two sensors in the WSN can communicate with each other either directly or via other sensors.

In this paper, we study the sensor placement problem to achieve both coverage and connectivity based on the confident information coverage model. We apply the grid approach to discretize the sensing field: the sensing field is divided into square grid cells, each with equal unit area, and sensors can be only placed at the center of each grid cell, which is called a candidate location. Our objective is to place the minimum number of sensors to form a connected network and to provide confident information coverage for all of the grid points. We formulate the sensor placement problem as a constrained optimization problem, which can be optimally solved by the exhaustive search algorithm. However, its time complexity increases exponentially with the number of candidate locations.

We propose two heuristic algorithms to find the approximate solutions for the sensor placement problem, namely the connected cover formation (CCF) algorithm and the cover formation and relay placement with redundancy removing (CFRP-RR) algorithm. The CCF algorithm constructs a connected cover for confident information coverage in a greedy manner: at each iteration, it places a new sensor at an unoccupied candidate location to cover the maximum number of uncovered grid points, and this newly deployed sensor must be connected with the already deployed sensor network. The CFRP-RR algorithm first places some sensors to cover all of the grid points in a greedy manner without considering network connectivity. After satisfying the coverage requirement, it then checks whether the deployed sensors can form a connected network. If not, it places some relays to form a connected network. After satisfying the connectivity requirement, the CFRP-RR algorithm includes redundancy removal as its final phase. As a relay may also contribute to coverage, there may exist some redundant sensors, which can be removed without compromising the coverage and connectivity requirement. In the final phase of CFRP-RR, it examines the coverage and connectivity requirements for all of the deployed sensors, and those redundant sensors are removed one by one in a greedy manner. The simulation results validate their effectiveness, and the CCF algorithm performs slightly better than the CFRP-RR algorithm.

The rest of the paper is organized as follows. Section 2 reviews some related work. In Section 3, we first briefly introduce the confident information coverage model and then formulate the sensor placement problem as a constrained optimization problem. Section 4 presents two heuristic algorithms, and the simulation results are provided in Section 5. Section 6 concludes the paper.

## 2. Related Work

Besides the simplest disk coverage model [9–12], many complicated sensor coverage models have been proposed for different applications in the literature. Some have taken coverage via the collaboration of sensors into consideration, such as the detection coverage model [13,14] and the estimation coverage model [15,16]. In [13], Wang *et al.* propose a probabilistic coverage model based on value fusion for target detection applications. In [15], Wang *et al.* propose an estimation coverage model, where sensors cooperate to make an estimate or decision for the data to sense at a particular location. Compared to the disk coverage model, they consider the information processing paradigm via the collaboration of sensors. However, this is based on a very simple single-valued parameter model and does not consider the spatial correlation of physical phenomena.

In WSNs, the sensor placement problem to achieve both coverage and connectivity has been extensively studied in recent years [17–26]. In [17], Ammari *et al.* consider sensing coverage phase transition (SCPT) and network connectivity phase transition (NCPT) problems using the percolation theory, and the correlated disk model is proposed to provide a basis for solving the SCPT and NCPT problems together. In [18,19], Bai *et al.* consider the optimal deployment pattern problem for different coverage and connectivity requirements in WSNs, and the optimal deployment patterns for all ranges of *r_c_*/*r_s_* to achieve full coverage and *k*-connectivity (*k* ≤ 6) are concluded in [20]. In [21], Cheng *et al.* compute the minimum number of relay sensors, such that the induced topology by all sensors is globally connected, which is modeled by the network optimization problem named Steiner minimum tree with minimum number of Steiner points and bounded edge length (SMT-MSP). In [22], Lloyd *et al.* investigate the single-tiered relay node placement (1tRNP) problem and present a simple minimum spanning tree (MST)-based approximation algorithm for 1tRNP. In [25], Natalizio *et al.* develop a mathematical model for determining the best placement of nodes by taking into consideration the energy of each node involved in the data flow. Additionally, in [26], Guerriero *et al.* propose and define different innovative optimization models by taking into account different performance objectives, and an extensive computational phase is carried out in order to assess the behavior of the developed models in terms of solution quality and computational effort.

In some practical situations, the monitored field has an irregular shape, which is not suitable for the optimal deployment pattern. Usually, the grid approach is applied to divide the irregular field into grid points in the literature [27-29], and full coverage can be achieved if all of the grid points can be covered. In [27], Chakrabarty *et al.* present novel grid coverage strategies for effective surveillance and target location in distributed sensor networks and formulate the minimum-cost sensor placement problem as an integer linear programming (ILP) problem. Ke *et al.* prove that the problem of deploying sensors on grid points to construct a WSN that fully covers critical grids using minimum sensors is NP-complete in [28], and the Steiner tree-based critical grid covering algorithm (STBCGCA) is proposed to solve this problem in [29].

For a randomly-deployed WSN with redundant sensors, Deng *et al.* have studied how to schedule one-modesensors and multi-modal sensors for confident information coverage to prolong the network lifetime in [30,31]. To the best of our knowledge, we are the first one to study the sensor placement problem to achieve both coverage and connectivity based on the confident information coverage model.

## 3. Problem Formulation

### 3.1. Confident Information Coverage

We first briefly review the CIC model proposed in our previous study [8], which is based on the theory of field reconstruction. Sensors are deployed within the sensing field to sample the attribute values of physical phenomena, and field reconstruction is used for their sampling values to interpolate or estimate the attribute values of physical phenomena at those unsampled locations.

Let *z^t^*(*x*) denote the true attribute value at a reconstruction point *x* at time *t*, *ẑ^t^*(*x*) its reconstructed (estimated) attribute value and *z^t^*(*s*) the attribute value sampled by a sensor *s*. A reconstruction function can be described as *f* : {*z^t^*(*s_i_*)|*s_i_* ∈ *S*(*x*)} → *ẑ^t^*(*x*), where *S*(*x*) denotes the set of sensors within the reconstruction area of *x*. The purpose of field reconstruction is to minimize the estimation error |*z^t^*(*x*) − *ẑ^t^*(*x*)|. Obviously, the estimation error |*z^t^*(*x*) − *ẑ^t^*(*x*)| is a random variable, whose probability distribution is unknown, as the physical phenomenon is a temporal-spatial process. Therefore, we use the time-averaged root mean square error (RMSE) to evaluate the reconstruction quality for each reconstruction point *x*, that is,

(1) $$\Phi \left(x\right)\equiv \sqrt{\frac{1}{T}\sum_{t=1}^{T}{\left(z^{t}\left(x\right)-{\hat{z}}^{t}\left(x\right)\right)}^{2}}$$

Based on the definition of RMSE, we give the definition of confident information coverage as follows:

Confident information coverage (Φ-coverage): Given a reconstruction function *f*, a space point *x* is called confident information covered (or Φ-covered) if the time-average RMSE of its reconstructed information Φ(*x*) is not larger than the application requirement *ϵ*, *i.e.*, Φ(*x*) ≤ *ϵ*. A sensing field is said completely Φ-covered if all of the space points within the sensing field are Φ-covered.

In this paper, we apply the ordinary kriging as the reconstruction function, as it only requires the physical phenomenon to be a second-order stationary process [32,33]. In spatial statistics, the variogram is a function describing the degree of spatial dependence of a spatial process [34]. In precision agriculture, the Gaussian variogram model has been widely used to describe most soil properties, such as soil temperature, humidity and fertility The Gaussian variogram is defined by $\gamma \left(h\right)={\text{C}}_{0}+{\text{C}}_{1}\left(1-e^{-\frac{h^{2}}{a^{2}}}\right)$ for *h* > 0 and *γ*(*h*) = 0 for *h* = 0, where C_0_ and C_1_ are called nugget and still, respectively, a is a constant related to the correlation range *D*, that is $D=\sqrt{3}a$, and the reconstruction area of *x* is exactly a disk centered at *x* with the radius of *D*. Note that a reconstruction point *x* only uses the attribute values sampled by those sensors within its reconstruction area for kriging. Without loss of generality, we let C_0_ = 0 and C_1_ = 1 in this paper. Hence, the Gaussian variogram is simplified as:

(2) $$\gamma \left(h\right)=1-e^{-\frac{h^{2}}{a^{2}}}$$

For a reconstruction point *x*, the ordinary kriging uses the weighted mean of the attribute values sampled by those sensors within the correlation range of *x* to interpolate or estimate the attribute value at *x*,

(3) $${\hat{z}}^{t}\left(x\right)=\sum_{i=1}^{n}\lambda_{i}z^{t}\left(s_{i}\right),s_{i}\in S\left(x\right)$$

where *n* = |*S*(*x*)| and *λ_i_*'s are the interpolation weights. According to the unbiased property of the ordinary kriging, the sum of weights is equal to one, *i.e.*, $\sum_{i=1}^{n}\lambda_{i}=1$. The optimal weights are obtained by minimizing the kriging variance. Using a Lagrange multiplier *μ*(*x*) for the minimization yields a linear kriging system of *n* + 1 equations with *n* + 1 unknowns,

(4) $$\{\begin{array}{l} \begin{array}{c} \begin{array}{cc} \sum_{j=1}^{n}\lambda_{j}\gamma \left(s_{i},s_{ij}\right)+\mu \left(x\right)=\gamma \left(s_{i},x\right), & i=1,2,\ldots ,n \end{array} \end{array}\\ \sum_{j=1}^{n}\lambda_{i}=1 \end{array}$$

where *γ*(*x*, *y*) = *γ*(*d*(*x*, *y*)) is the Gaussian variogram of the monitored physical phenomenon and *d*(*x*, *y*) is the Euclidean distance between *x* and *y*. After some algebra, Φ(*x*) can be computed by:

(5) $$\Phi \left(x\right)=\sqrt{\sum_{i=1}^{n}\lambda_{i}\gamma \left(s_{i},x\right)+\mu \left(x\right)}$$

where:

(6) $$\left(\begin{array}{c} \lambda_{1}\\ \vdots \\ \lambda_{n}\\ \mu \left(x\right) \end{array}\right)={\left(\begin{array}{cccc} \gamma \left(s_{1},s_{1}\right) & \ldots & \gamma \left(s_{1},s_{n}\right) & 1\\ \vdots & \ddots & \vdots & \vdots \\ \gamma \left(s_{n},s_{1}\right) & \ldots & \gamma \left(s_{n},s_{n}\right) & 1\\ 1 & \ldots & 1 & 0 \end{array}\right)}^{-1}\times \left(\begin{array}{c} \gamma \left(s_{1},x\right)\\ \vdots \\ \gamma \left(s_{n},x\right)\\ 1 \end{array}\right)$$

Equation (3) implies that the interpolation can be done via the collaboration of sensors within the reconstruction area. It is also possible that we use only one sensor closest to the unsampled location for interpolation. In this case, Φ(*x*) can be computed by:

(7) $$\Phi \left(x\right)=\sqrt{2\gamma \left(s_{1},x\right)}=\sqrt{2\left(1-e^{-\frac{r^{2}}{a^{2}}}\right)}$$

where r = *d*(*s*_1_, *x*). Obviously, Φ(*x*) is positive correlated with *r*, and for any *r′* ≤ *r*, Φ(*x′*) ≤ Φ(*x*), where *r′* = *d*(*s*_1_, *x′*). Therefore, the CIC model is downward compatible with the disk coverage model.

**Figure 1:**
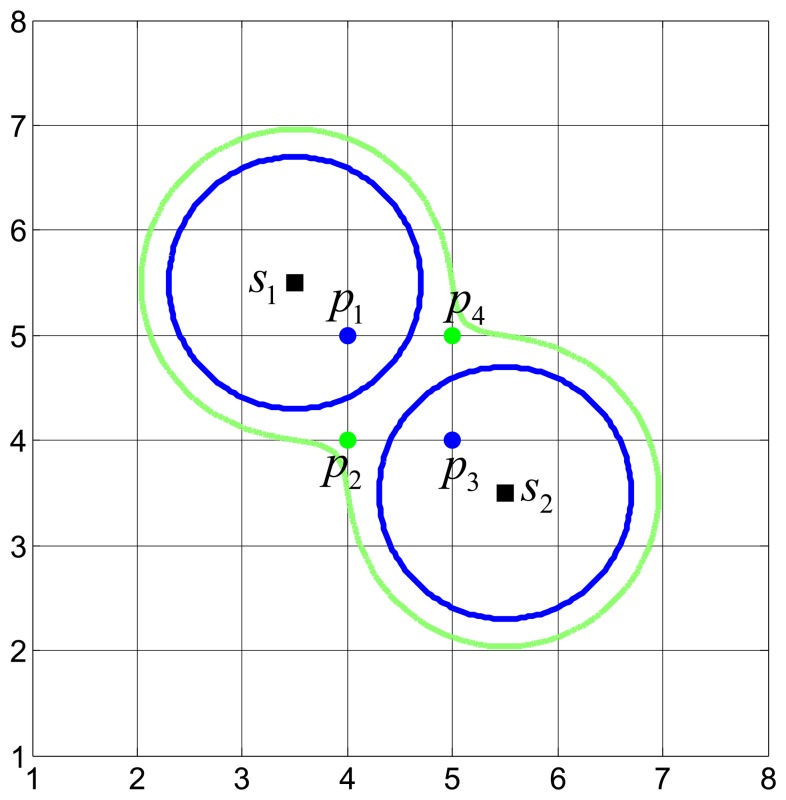
Illustration of the disk coverage model and the confident information coverage (CIC) model.

Figure 1 plots the disk coverage model of two individual sensors and the CIC model via the collaboration of two sensors. In Figure 1, *s*_1_, *s*_2_ are two sensors (the squares dots) and *p*_1_, *p*_2_, *p*_3_, *p*_4_ are four grid points (the circular dots). Two blue disks are the covered area of *s*_1_ and *s*_2_, respectively. The green dumbbell-shaped area is the Φ-covered area via the collaboration of *s*_1_ and *s*_2_. Due to the inclusion of the collaboration of sensors, the CIC model extends the disk coverage model.

The CIC model can be used in many monitoring applications, such as precision agriculture applications [5,6]. Specifically, when we want to monitor the soil temperature on a farm, we can deploy some sensors at some locations to capture the temperature at these locations. As the number of deployed sensors is limited, we can only obtain the temperature of some discrete points. In order to monitor the continuous soil temperature distribution, we can apply the theory of field reconstruction for estimating/interpolating soil temperature values at unsampled locations. Furthermore, in most cases, a physical phenomenon, like soil temperature, within a continuous space can be modeled as a spatial stochastic process with some spatial correlation. With the help of this spatial correlation, we can reconstruct the temperature in the continuous space by making use of the temperature captured by sensors located at some discrete points.

### 3.2. Sensor Placement Problem

Like many existing works [27–29], we also apply the grid approach to discretize the sensing field. In particular, the sensing field is divided into square grid cells, each with equal area, and sensors can be only placed at the center of each grid cell, which is called a candidate location. We use all of the grid points to represent the sensing field, that is to say, the sensing field is Φ-covered, if all of the grid points are Φ-covered.

As illustrated in Figure 1, in the disk coverage model, only *p*_1_ and *p*_3_ can be covered by *s*_1_ and *s*_2_, respectively, while *p*_2_ and *p_4_* cannot be covered. In the CIC model, *p*_1_ and *p*_3_ can be Φ-covered by *s*_1_ and *s*_2_, respectively, while *p*_2_ and *p*_4_ can also be Φ-covered via the collaboration of *s*_1_ and *s*_2_.

We assume that there are *I* candidate locations and *J* grid points in the sensing field, and we use subscripts *i* and *j* to index a candidate location and a grid point, respectively Let *x_i_* indicate whether a sensor is placed at *i*, that is,

(8) $$x_{i}=\{\begin{array}{ll} 1, & \text{a sensor is placed at}\;i\\ 0, & \text{otherwise} \end{array}$$

Let *δ_j_* indicate whether a grid point is Φ-covered, that is,

(9) $$\delta_{j}=\{\begin{array}{ll} 1, & j\;\text{is}\;\Phi -\text{covered}\\ 0, & \text{otherwise} \end{array}$$

Our objective is to place the least number of sensors to form a connected network to provide confident information coverage for all of the grid points, which can be formulated as the following constrained optimization problem:

(10) $$\text{minimize}\sum_{i=1}^{I}x_{i}$$

subject to

(11) $$\sum_{j=1}^{J}\delta_{j}=J$$

Equation (11) ensures that all of the grid points have been Φ-covered. At the same time, all of the sensors must form a connected network. Compared to the grid coverage based on the unit disk model, our problem is more complicated due to the fact that in the CIC coverage, a grid point can be cooperatively covered by sensors not within its disk range.

The exhaustive search algorithm can be used to optimally solve the sensor placement problem by trying all of the possible placements. Among all of the possible placements, it finds the optimal one with the least number of sensors to achieve both coverage and connectivity. If there are *I* candidate locations, the number of all of the possible placements is $\sum_{i=1}^{I}C_{I}^{i}=2^{I}-1$. Obviously, the time complexity of the exhaustive search algorithm increases exponentially with the number of candidate locations. When the problem instance is large, the computation of the exhaustive search algorithm will be a very time-consuming process. We next propose two heuristic algorithms to find the approximate solutions for the sensor placement problem, yet with significantly reduced time complexity.

## 4. Solutions for The Placement Problem

We propose two heuristic algorithms to find the approximate solutions for the sensor placement problem, namely the connected cover formation (CCF) algorithm and the cover formation and relay placement with redundancy removing (CFRP-RR) algorithm. Both of them iteratively place a new sensor at each step, until the coverage and connectivity requirements can be satisfied.

We next summarize some symbols used in these two algorithms as follows:

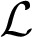
/ 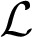
*_u_*: the set of all/unoccupied candidate locations.

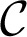
: the set of deployed sensors, *i.e.*, the set of selected candidate locations.
- *R_c_*: the communication range of a sensor.

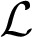
*_a_*: the set of available candidate locations, which is defined by:
  (12) $$L_{a}=\left\{i|i\in L_{u},\;\text{and}\;\exists c\in C,d\left(i,c\right)\leq R_{c}\right\}$$
- i*: the selected candidate location to place a sensor at each iteration.

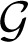
/ 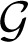
*_u_*: the set of all/uncovered grid points.

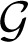
(*i*): the set of newly covered grid points due to the placement of sensor *i*.

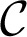
(*j*): the set of deployed sensors within the correlation range of grid point *j*.
- *C_j_*: the working GPC of grid point *j*.
- *G*(*V*, *E*, *W*): the undirected weighted complete graph on the vertices in *V*, where for two vertices *u*, *v* ∈ *V*, the weight of the edge (*u, v*) is equal to their distance, *i.e.*, *w*(*u*, *v*) = *d*(*u*, *v*).

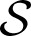
(*G*, *R_c_*): the set of subgraphs of *G* by removing those edges with weight larger than *R_c_*.
- *G_k_*_:_ the *k*-th subgraph in 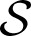
(*G*, *R_c_*), *k* = 1, 2, …, *K*.
- *D_min_*((*u*, *G_k_*),(*v*, *G_l_*)): the shortest subgraph distance between two subgraphs *G_k_* and *G_l_*, *i.e.*, the shortest distance between vertex *u* ∈ *G_k_* and vertex *v* ∈ *G_l_*.

### 4.1. The Connected Cover Formation Algorithm

The CCF algorithm places a sensor at each iteration to form a connected sensor cover in a greedy manner. First, 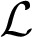
*_a_* = 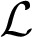
*_u_* = 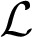, the candidate location *i* ∈ 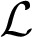
*_a_* with the largest size of 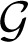
(*i*), denoted by i*, is selected to place a sensor to cover the largest number of grid points in 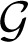
*_u_*. Then, i* is removed from 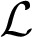
*_u_*, and 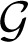
(i*) is also removed from 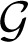
*_u_*. Then, we reconstruct 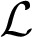
*_a_* according to Equation (12), which indicates that each element in 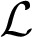
*_a_* is an unoccupied candidate location, and its distance to at least one occupied candidate location is not greater than *R_c_*. At the next iteration, another candidate location i* ∈ 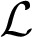
*_a_* is selected to place a sensor to cover the largest number of grid points in 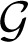
*_u_*. As we do not differentiate how a grid point is Φ-covered, maximizing the number of newly covered grid points at each iteration is equivalent to maximizing the number of total covered grid points, *i.e.*, maximize $\sum_{j=1}^{J}\delta_{j}$. The above process repeats, until all of the grid points have been Φ-covered by the deployed sensors, *i.e.*, $\sum_{j=1}^{J}\delta_{j}=J$. Furthermore, as we only place such a sensor that connects to the already deployed sensor network at each iteration, the final deployed sensor network is guaranteed to be a connected network.

The pseudo-codes of the CCF algorithm are provided in Algorithm 1. The inner while loop (Lines 9 to 16) is to find a group of sensors from the set of deployed sensors within the correlation range of grid point *j* to Φ-cover *j*. Note that this is done in a greedy manner by selecting a sensor c with the greatest contribution to decrease the RMSE of *j*. The inner for loop (Lines 6 to 17) is to count the number of newly covered grid points due to the placement of sensor *i* from the available candidate locations. The outer for loop (Lines 4 to 21) is to place a sensor to cover the largest number of uncovered grid points. The outer while loop (Lines 2 to 24) terminates, if all of the grid points have been Φ-covered by the deployed sensors.


**Algorithm 1** The connected cover formation (CCF) algorithm.
**Input:**The set of all candidate locations 

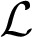
, the set of all grid points 

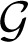
**Output:**The set of deployed sensors 

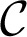
1:

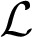
*_a_* = 

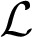
*_u_* = 

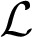
, 

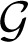
*_u_* = 

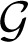
, 

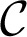
 = ∅2:**while**

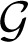
*_u_* = ∅ **do**3: *N_max_* = −*inf*, 

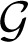
*_new_* = ∅;4: **for** each *j* ∈ 

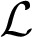
*_a_*
**do**5:  *N* = 0, 

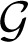
(*i*) = ∅;6:  **for** each *j* ∈ 

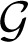
*_u_*
**do**7:   *C_j_* = ∅;8:   Construct 

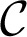
(*j*) from 

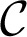
∪{*i*} based on *j*;9:   **while**

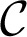
(*j*) = ∅ **do**10:    Select *c* ∈ 

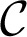
(*j*) with the greatest contribution to decrease the RMSE of *j*;11:    *C_j_* = *C_j_*∪{*c*}, 

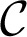
(*j*) = 

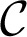
(*j*)\ {*c*};12:    **if**
*j* is Φ-covered by *C_j_*
**then**13:     *N* = *N* + 1, 

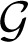
(*i*) = 

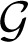
(*i*)∪{*j*};14:     **break;**15:    **end if**16:   **end while**17:  **end for**18:  **if**
*N* > *N_max_*
**then**19:   *N_max_* = *N*, i* = *i*, 

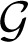
*_new_* = 

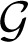
(*i*);20:  **end if**21: **end for**22:  

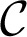
 = 

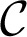
∪{i*}, 

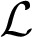
*_u_* = 

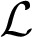
*_u_*\{i*}, 

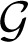
*_u_* = 

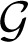
*_u_*\

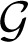
*_new_*;23: Construct a new 

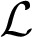
*_a_* from 

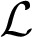
*_u_* based on 

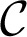
;24:**end while**


There are four loops in CCF: the inner while loop (Lines 9 to 16), the inner for loop (Lines 6 to 17), the outer for loop (Lines 4 to 21) and the outer while loop (Lines 2 to 24), which need *I*, *J*, *I* and *J* times computations at most, respectively. Therefore, the time complexity of CCF is *O*(*I*^2^ × *J*^2^).

### 4.2. The Cover Formation and Relay Placement with Redundancy Removal Algorithm

The CFRP-RR algorithm consists of three phases: the first phase aims to achieve complete coverage; the second phase aims to achieve connectivity; and the third phase aims to remove redundant sensors. In the first phase, CFRP-RR places one sensor at each iteration to maximize the number of grid points that are Φ-covered, until all grid points are Φ-covered. The main difference to CCF is that a new sensor can be placed at any unoccupied candidate location in CFRP-RR, rather than any available candidate locations in CCF. Recall that 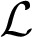
*_a_* ⊂ 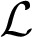
*_u_*. Selecting i* from 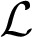
*_a_* guarantees that the deployed sensors form a connected network, but selecting i* from 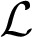
*_u_* cannot guarantee a connected network.

After the first phase, if the deployed sensors cannot form a connected network, then in the second phase, CFRP-RR places some sensors as relays to connect those isolated subnetworks. Specially, we first construct an undirected weighted complete graph *G*(*V*, *E*, *W*) by setting *V* = 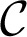, and the weight of an edge (*u*, *v*) ∈ *E* is equal to the distance between *u* and *v*. Then, we construct the set of subgraphs 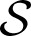
(*G*, *R_c_*) by removing those edges in *G* with weight larger than *R_c_*. Suppose there are *K* subgraphs in *S*, denoted by *G*_1_, *G*_2_, …, *G_K_*. Note that *K* = 1 implies that the deployed sensors form a connected network. Obviously, sensors in the same subgraph are connected, and sensors in different subgraphs are disconnected.

For any two disconnected subgraphs *G_k_* and *G_l_* in 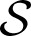, we compute the distances between vertices of *G_k_* and vertices of *G_l_*. Among all of the distances, we call the minimum distance the shortest subgraph distance (SSD) between *G_k_* and *G_l_*. Note that there are *K* subgraphs, so we obtain *K*(*K* − 1)/2 such SSDs in total, and the minimum SSD, denoted by *D_min_*((*u*, *G_k_*), (*v*, *G_l_*)), is selected for relay placement. We then use a furthest progress method to place relays to connect *u* and *v*. After *G_k_* and *G_l_* have been connected by the newly placed relays, we obtain a new set of deployed sensors. Then, we repeat the above process to examine network connectivity and to connect disconnected subgraphs, until 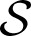
(*G*, *R_c_*) contains only one element (*K* = 1), then the deployed sensors form a connected network.

**Figure 2:**
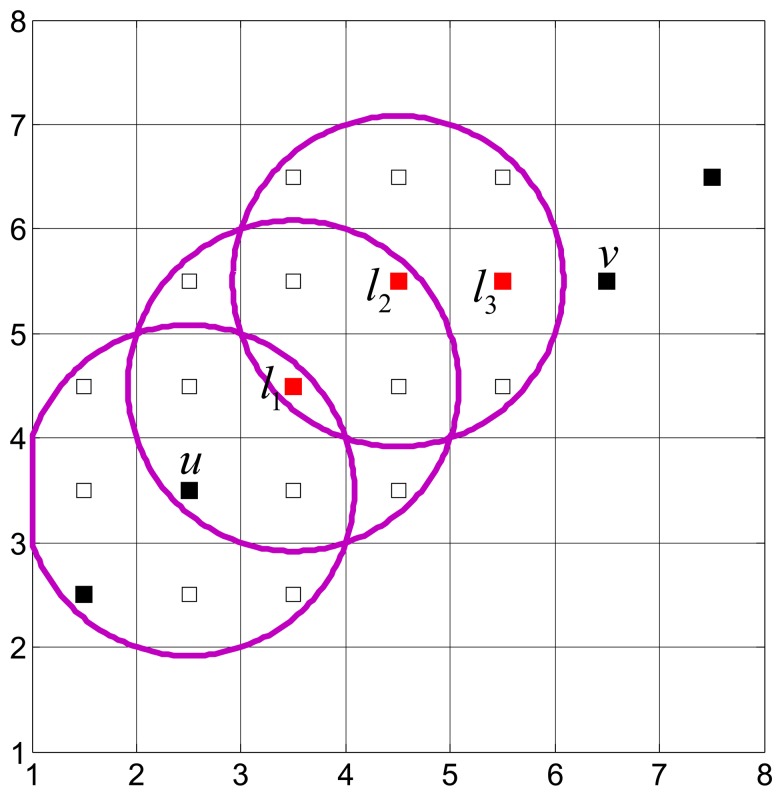
The process of relay node placement.

We next explain the furthest progress method for relay placement by an example. As shown in Figure 2, we need to place relay(s) to connect *u* and *v*. Starting from *u*, there are seven unoccupied candidate locations within the communication range of u (namely, the disk centered at *u* with a radius of *R*_c_), and *l*_1_ is the closest one to *v*. That is, *l*_1_ is the furthest progress from *u* to *v*. Therefore, *l*_1_ is selected to place a relay. If *d*(*l*_1_, *v*) > *R_c_*, then starting from *l*_1_ we repeat the above process, until *u* and *v* are connected via the newly deployed relay(s). In Figure 2, three relays are deployed to connect *u* and *v*, namely *l*_1_, *l*_2_ and *l*_3_.

We omit the pseudo-codes of CFRP-RR Phase 1, as they are similar to those of CCF, except for using 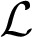
*_u_* instead of 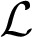
*_a_* at each iteration. The pseudo-codes of CFRP-RR Phase 2 are provided in Algorithm 2. The function *ComputeSSD* is to compute the SSD between two subgraphs, and the minimum SSD is obtained by comparing all of the SSDs (Lines 4 to 12). The function *PlaceRelay* is to place relays according to the furthest progress method, which returns the new set of deployed sensors (including relays). The outer while loop (Lines 3 to 17) is to connect two subgraphs at each iteration, until all of the deployed sensors form a connected network.


**Algorithm 2** The cover formation and relay placement with redundancy removing (CFRP-RR) algorithm, Phase 2.
**Input:** The set of deployed sensors 

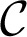
, the communication range *R_c_*;**Output:** The new set of deployed sensors 

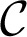
1:Construct *G*(*V*, *E*, *W*) based on 

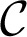
;2:Construct 

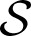
(*G*, *R_c_*) based on *G* and *R_c_*;3:**while** |

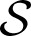
(*G*, *R_c_*)| > 1 **do**4: *D_min_* = inf, u* = v* = inf;5: **for** each *G_k_* ∈ 

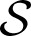

**do**6:  **for** each *G_l_* ∈ 

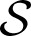

**and**
*G_k_* ≠ *G_l_*
**do**7:   *d*(*u*, *v*) = *ComputeSSD*(*G_k_*, *G_l_*);8:   **if**
*d*(*u*, *v*) < *D_min_*
**then**9:    *D_min_* = *d*(*u*, *v*), u* = *u*, v* = *v*;10:   **end if**11:  **end for**12: **end for**13:  

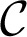
*_relay_* = *PlaceRelay* (u*, v*);14:  

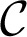
 = 

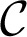
 ∪ 

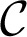
*_relay_*;15: Construct *G*(*V*, *E*, *W*) based on 

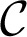
;16: Construct 

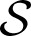
(*G*, *R_c_*) based on *G* and *R_c_*;17:**end while**


After the second phase, both complete coverage and connectivity have been achieved. Note that the deployed relays are actually a sensor, which can also participate in coverage, so there may exist some redundant sensors for coverage, and we can further remove them without compromising the coverage and connectivity requirements. In the third phase, CFRP-RR removes one redundant sensor at each iteration in a greedy manner, until no redundant sensor can be found.

A sensor *r* is called redundant if, after removing it, the rest of the sensors in 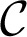
^¬^*^r^* ≡ 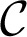
\{*r*} can still cover all grid points and form a connected network. It is possible that there exist multiple redundant sensors in 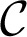, and we cannot remove all of them at the same time to avoid breaching the coverage and connectivity requirements. In the third phase, we remove the redundant sensors one by one at each iteration according to their redundancy weights.

Suppose that a redundant sensor *r* is removed; there may still exist redundant sensors in 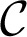
^¬^*^r^*. We define the redundancy weight of a redundant sensor *r* as the number of redundant sensors in 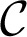
^¬^*^r^*, and the redundant sensor with the largest redundancy weight is removed at each iteration. Then, we repeat the above process to examine the redundancy for the rest of the sensors, until no redundant sensor can be found.

The pseudo-codes of CFRP-RR Phase 3 are provided in Algorithm 3. The function *computeRedundantSensor* is to obtain the set of redundant sensors. The for loop (Lines 4 to 10) is to find the redundant sensor with the largest redundancy weight. The while loop (Lines 2 to 13) is to remove redundant sensors one by one at each iteration, until no redundant sensor can be found.


**Algorithm 3** The CFRP-RR algorithm, Phase 3.
**Input:** The set of deployed sensors 

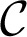
, the set of grid points 

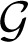
, the communication range *R_c_***Output:** The new set of deployed sensors 

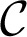
;1:

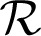
 = *ComputeRedundantSensor*(

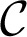
, 

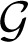
, *R_c_*);2:**while**

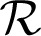
 = ∅ **do**3: *W_max_* = −inf;4: **for** each *r* ∈ 

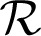

**do**5:  

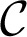
^¬^*^r^* = 

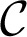
\{*r*}6:  

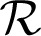
^¬^*^r^* = *ComputeRedundantSensor*(

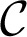
^¬^*^r^*, 

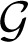
, *R_c_*);7:  **if |

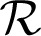
**^¬^*^r^*| > *W_max_*
**then**8:   *W_max_* = |

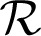
^¬^*^r^*|, r* = *r*;9:  **end if**10: **end for**11: 

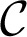
 = 

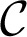
\{r*};12: 

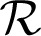
 = *ComputeRedundantSensor*(

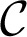
, 

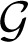
, *R_c_*);13:**end while**


Similar to the analysis in CCF, the time complexity of CFRP-RR Phase 1 is *O*(*I*^2^ × *J*^2^). There are three loops in CFRP Phase 2: the inner for loop (Lines 6 to 11), the outer for loop (Lines 5 to 12) and the while loop (Lines 3 to 17), which all need *I* times computations at most, so the time complexity of CFRP-RR Phase 2 is *O*(*I*^3^). There are two loops in CFRP-RR Phase 3: the for loop (Lines 4 to 10) and the while loop (Lines 2 to 13), which all need *I* times computations at most, so the time complexity of CFRP-RR Phase 3 is *O*(*I*^2^). Then, the total time complexity of CFRP-RR is *O*(*I*^2^ × *J*^2^) + *O*(*I*^3^) + *O*(*I*^2^) = *O*(*I*^2^ × max{*I*, *J*^2^}). Note that, in general, *I* < *J*^2^, so the time complexity of CFRP-RR is *O*(*I*^2^ × *J*^2^).

## 5. Simulation Results

In this section, we use simulations to evaluate the performance the CCF algorithm and the CFRP-RR algorithm. In addition, we compare them with the minimum spanning tree (MST)-based node deployment in Relay Node Placement in Wireless Sensor Networks and the classical simulated annealing (SA) algorithm.

The basic idea of the MST algorithm is as follows. First, some sensors are deployed to achieve complete coverage. Second, we construct a graph based on all of the deployed sensors, and the weight of the edge is exactly the distance of two sensors. Third, we build a minimum spanning tree (MST) based on this graph. Fourth, we examine all of the edges in this MST, and for those edges whose weights are larger than the communication range, we deploy some relays to connect them. Finally, we can obtain a network achieving both coverage and connectivity. The basic idea of the SA algorithm is as follows. First, all of the candidate locations are each occupied by one sensor, and the network can achieve both coverage and connectivity. Second, at each iteration, we remove one sensor according to a given rule, and the above process repeats, until no sensor can be removed without compromising the coverage and connectivity requirements. Finally, we can also obtain a network achieving both coverage and connectivity.

We consider a square monitored field and divide it into in total *M* × *M* grid cells, each with equal unit area. Therefore, there are in total (*M* + 1)^2^ grid points to be Φ-covered and *M*^2^ candidate locations to place sensors. Specially, we consider a monitored field with 11^2^ = 121 grid points and 10^2^ = 100 candidate locations. First, we compare these four algorithms for the following three parameters:

- *ϵ*: the RMSE threshold of confident information coverage.
- *D*: the correlation range of a physical phenomenon.
- *R_c_*: the communication range of a sensor.

**Figure 3:**
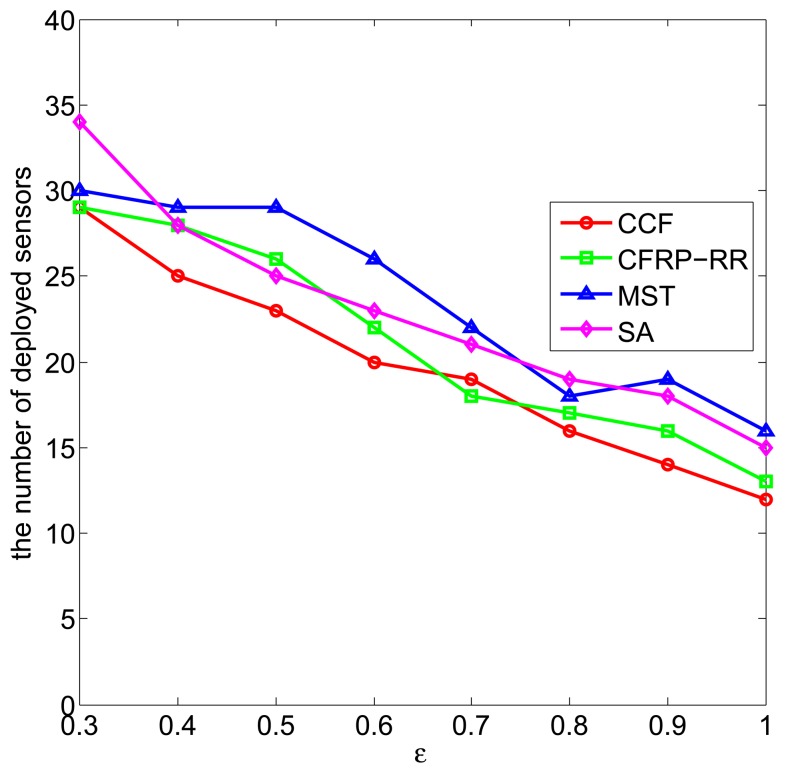
The number of deployed sensors *vs. ϵ*.

Figure 3 plots the number of deployed sensors against *ϵ*, when *D* = 5, *R_c_* = 2.5, *M* = 10 and *ϵ* ranges from 0.3 to one. It can be observed that with the increase of *ϵ*, the number of deployed sensors decreases, which is due to the fact that, when the application requirement *ϵ* increases, the already deployed sensors can cover a larger area of the monitored field, and less new sensors are required to be deployed to satisfy the coverage requirement. In addition, we can observe that the CCF algorithm performs slightly better than the CFRP-RR algorithm, and both of them perform better than the MST algorithm and the SA algorithm.

**Figure 4:**
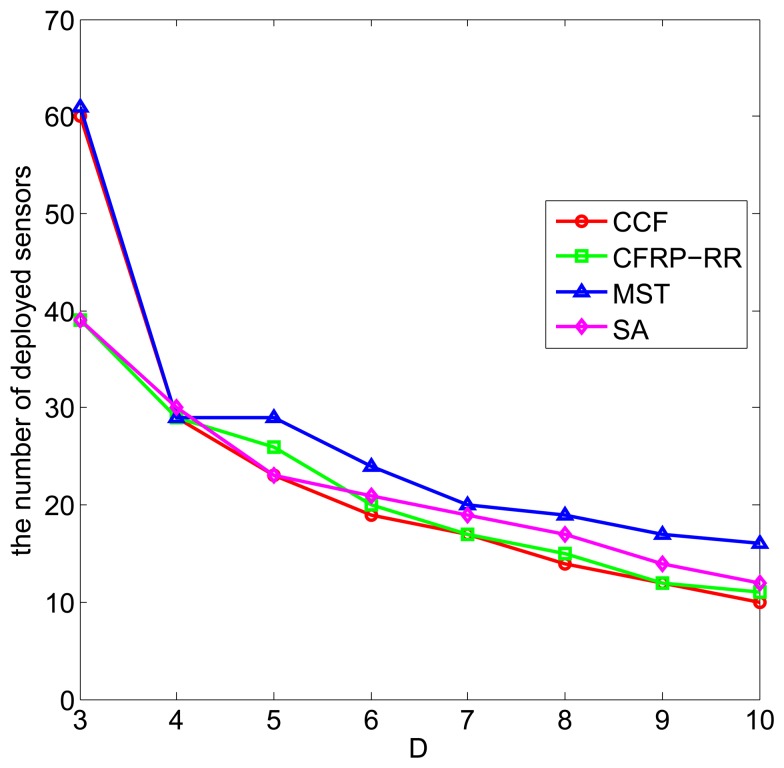
The number of deployed sensors *vs. D*.

Figure 4 plots the number of deployed sensors against *D*, when *ϵ* = 0.5, *R_c_* = 2.5, *M* = 10 and *D* ranges from three to 10. It can be observed that with the increase of *D*, the number of deployed sensors decreases, which is due to the fact that, when the correlation range *D* increases, more already deployed sensors can participate to cover the grid points in the monitored field and less new sensors are required to be deployed to satisfy the coverage requirement. In addition, we can observe that the CCF algorithm performs slightly better than the CFRP-RR algorithm, and both of them perform better than the MST algorithm and the SA algorithm.

**Figure 5:**
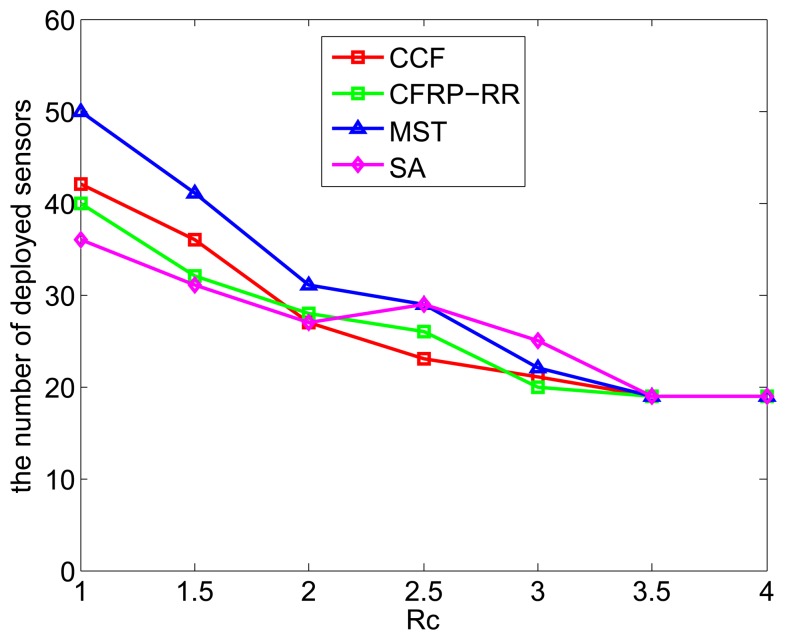
The number of deployed sensors *vs. R_c_*.

Figure 5 plots the number of deployed sensors against *R_c_*, when *ϵ* = 0.5, *D* = 5, *M* = 10 and *R_c_* ranges from one to four. It can be observe that with the increase of *R_c_*, the number of deployed sensors decreases, which is due to the fact that, with the increase of *R_c_*, less sensors are required to be deployed to satisfy the connectivity requirement. Again, the CCF algorithm performs slightly better than the CFRP-RR algorithm, and both of them perform better than the MST algorithm and the SA algorithm. In addition, when *R_c_* ≥ 3.5, all four curves become flat and coincide with each other, which is due to the fact that, when *R_c_* is sufficiently large, connectivity comes with coverage and is no longer an independent restrictive condition.

**Figure 6:**
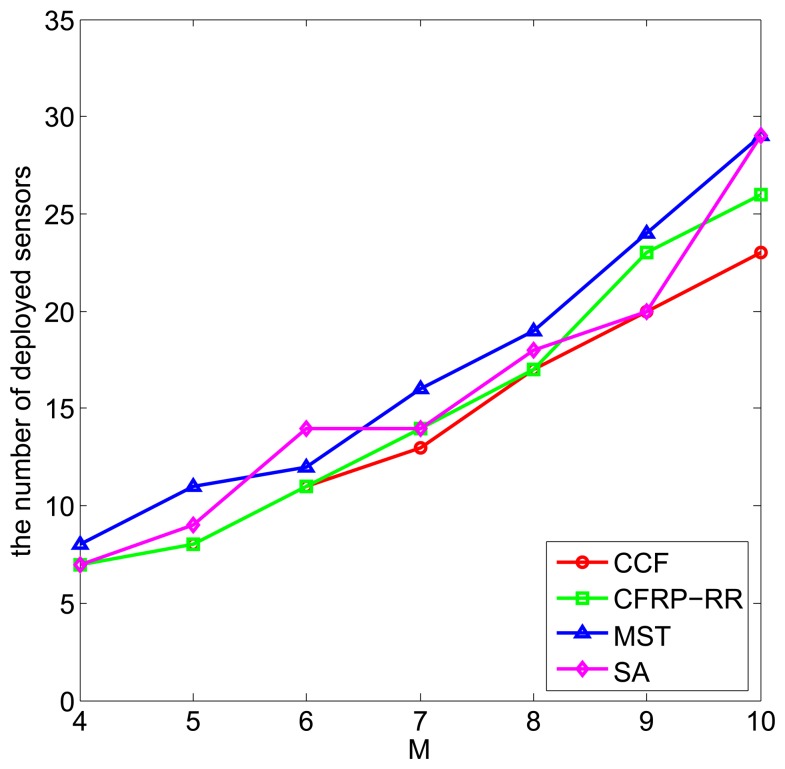
The number of deployed sensors *vs. M*.

Finally, we compare these three algorithms for different values of *M*. Figure 6 plots the number of deployed sensors against *M*, when *ϵ* = 0.5, *D* = 5, *R_c_* = 2.5 and *M* ranges from four to 10. It can be observed that with the increase of *M*, the number of deployed sensors increases, which is due to the fact that a larger area of the monitored field requires more sensors to be deployed to satisfy the coverage requirement. Still, the CCF algorithm performs slightly better than the CFRP-RR algorithm, and both of them perform better than the MST algorithm and the SA algorithm.

In summary, compared to two algorithm (the MST algorithm and the SA algorithm) in the literature, our two algorithms can achieve better performance for different parameters (*ϵ*, *D*, *R_c_*, *M*). Furthermore, the CCF algorithm performs better than the CFRP-RR algorithm. Both of them can provide two different ideas and methods to solve coverage and connectivity problems in WSN.

## 6. Conclusions

In this paper, we have studied the sensor placement problem to achieve both coverage and connectivity based on the confident information coverage model. We formulated the sensor placement problem as a constrained optimization problem and proposed two heuristic algorithms, namely the CCF algorithm and the CFRP-RR algorithm, to solve the sensor placement problem. The simulation results have validated their effectiveness, and the CCF algorithm performs slightly better than the CFRP-RR algorithm.
